# A scoping review on population-centered indicators for cancer care continuum

**DOI:** 10.3389/fpubh.2022.912946

**Published:** 2022-10-14

**Authors:** Vasuki Rajaguru, Jieun Jang, Jeoung A. Kwon, Jae Hyun Kim, Jaeyong Shin, Mison Chun

**Affiliations:** ^1^Department of Healthcare Management, Graduate School of Public Health, Yonsei University, Seoul, South Korea; ^2^Hinda and Arthur Marcus Institute for Aging Research, Hebrew SeniorLife, Harvard Medical School, Boston, Massachusetts, United States; ^3^Institute of Health Services Research, Yonsei University, Seoul, South Korea; ^4^Department of Health Administration, Dankook University, Cheonan, South Korea; ^5^Department of Preventive Medicine, College of Medicine, Yonsei University, Seoul, South Korea; ^6^Department of Radiation Oncology, School of Medicine, Ajou University, Suwon, South Korea

**Keywords:** cancer, indicators, cancer care, monitoring, quality improvement, population, review, health care

## Abstract

**Purpose:**

The purpose of this study was to develop prioritized cancer indicators and measure the population-based monitoring of the entire life cycle of cancer care, guiding the improvement of care delivery systems.

**Methods:**

Scoping review was performed based on the Joanna Briggs Institute's methodology. Electronic databases were searched in PubMed, Cochrane Library, EMBASE, Ovid Medline, RISS, KISS, and KoreaMed. The searches were limited to articles published in English between 2010 and 2020. No restrictions were applied regarding the publication status or country of origin, and all study designs were included. Gray literature was used to broaden the search's scope, identify new recommendations, need to be in connect with subject experts, and explore pertinent websites. The process and selected indicators were analyzed based on their frequency distribution and percentage.

**Results:**

The literature search yielded 6,202 works. In addition, national and international cancer guidelines were obtained from official database reports. A total of 35 articles and 20 reports regarding cancer indicators were finally selected for data synthesis. Based on them, 254 core sets of cancer indicators were identified. The selected indicators were classified into six domains based on the continuum of cancer care and survivor's life cycle, namely, primary prevention (61, 24.0%), secondary prevention (46, 18.1%), treatment (85, 33.5%), quality of care (33, 13.0%), survivor management (33, 13.0%), and end-of-life care (14, 5.5%).

**Conclusion:**

There is a growing interest in developing specific areas of cancer care. Cancer indicators can help organizations, care providers, and patients strive for optimal care outcomes. The identified indicators could guide future innovations by identifying weaknesses in cancer prevention and management.

## Introduction

Cancer is one of the leading causes of death in Korea, where 243,263 new cancer cases and 80,546 cancer deaths are expected in 2020 (1). Although the cancer incidence rates are anticipated to decrease slightly, the burden of most types of cancer will continue to grow as the population ages (2).

The Korea Central Cancer Registry is a nationwide, hospital-based cancer registry initiated by the Korean Ministry of Health and Welfare in 1980 (3). It compares the status of cancer care based on the 5-year relative survival rates. For instance, 5-year relative survival rate of Korea's cervical cancer is 76.8%, and that of colorectal cancer is 63.7%, the best among the member nations of the Organization for Economic Co-operation and Development (OECD). The nation's breast cancer survival rate is 82.2% like the OECD average (4).

**Figure 1:**
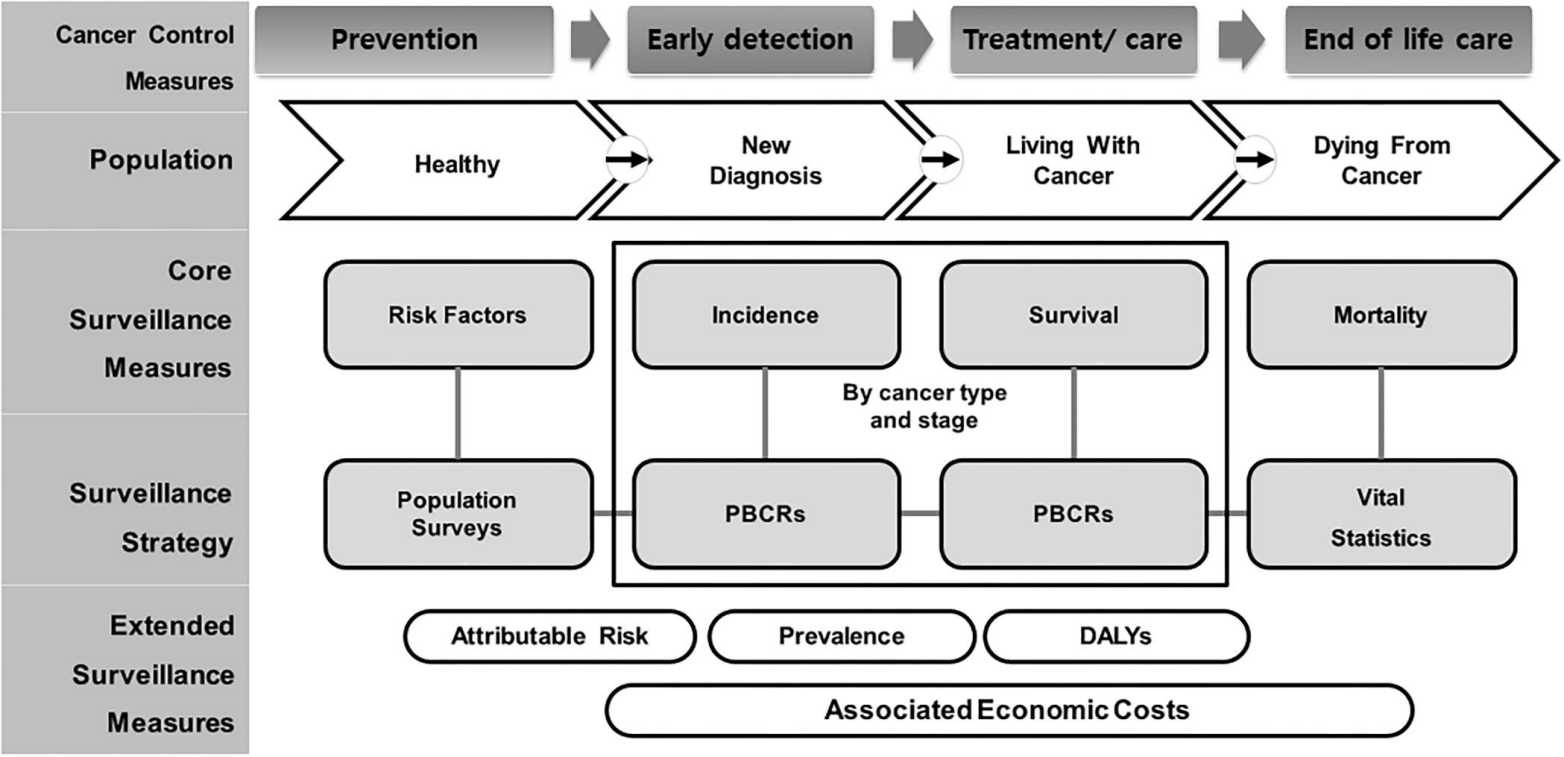
Conceptual framework of population-based cancer indicators and measurement for entire life cycle of cancer care level. Adapted from Piñeros et al. (5). PBCR, Population-Based Cancer Registry; DALYs, Disability-Adjusted Life-Years.

There are a growing number of studies related to cancer indicators. However, they focus on very specific aspects, such as incidence and mortality, special procedures or radiological treatment, and palliative care. Population-based cancer registries (PBCRs) complementing incidence and mortality by types of cancer have been collected, and it included as one of the 25 core indicators among non-communicable disease Global Monitoring Framework; the necessary technical support in planning and developing PBCRs in low-, middle-, and high-income countries is being provided through the International Association for Research on Cancer (IARC)-led partnership, the Global Initiative for Cancer Registry Development, and has presented a framework for managing patients with cancer based on the cancer life cycle, dividing it into four groups, namely, healthy population, early diagnosis, living with cancer, and dying (Figure 1) (5). Therefore, this review focused on developing cancer indicators as per the framework.

Several studies on the performance or quality indicators of cancer care have shown that the indicators are associated with an improvement in either patient satisfaction or care quality (5–7). Furthermore, many reports have focused on the development of effective therapies, the implementation of clinical practice guidelines, healthcare provision through multidisciplinary and interprofessional teams at all stages of the disease, and patient-centered care (6–8). There are limited studies reported regarding the population-based monitoring of cancer indicators that encompass the specific types of the cancer-care continuum (5, 8). However, minimal attention has been paid to the life cycle of the cancer or the cancer-care continuum. Many new approaches have also emerged. However, there is no study that focuses on the entire life cycle of cancer care. Therefore, the aim of this study was to develop a population-based monitoring of cancer indicators and measurement for the entire life cycle of cancer care.

Scoping reviews are widely used to draw a map and analyze the existing scientific evidence in complex or relatively unexamined fields. We, therefore, initially preferred to review and compare the perspectives and attributes of the national and international cancer care guidelines. The current scoping review sought to find, examine, and describe the scientific literature on cancer indicators related to the measurement of community-based monitoring for the span of cancer care. To the best of our knowledge, this is the first scoping review to examine population-based cancer monitoring for the entire life cycle of cancer.

The purpose of this study was to identify the existing scientific literature and international guidelines regarding cancer indicators for the population-based monitoring of the entire continuum of cancer care using a scoping review methodology as prior knowledge of our ongoing project. In addition, the set of review will be providing the key knowledge of population-based cancer indicator measures that support the planning and evaluation of cancer indicators across the cancer-care continuum by elaborating the unique part of our ongoing project. In addition, we will continue this study in order to demonstrate how to concise the selected indicators with proper measurement by the numerator and denominator rate in the future by a panel discussion.

## Methods

This scoping review followed the six steps of the Arksey and O'Malley (9) and was conducted in compliance with the Preferred Reporting Items for Systematic Reviews and Meta-Analyses (PRISMA) statement for scoping reviews (10). In addition, gray literature was performed to identify the existing national and international cancer indicators and guidelines.

### Research questions

By using the scoping review methodology, the following specific research questions have to be addressed:

1. “What are the national and international guidelines of cancer indicators guidelines, which focus community, based monitoring of cancer indicators? And What are the types of monitoring indicators in cancer management have been reported?”
2. “What/Which are the patient, interventions, and outcomes with measurement of cancer indicators are currently in use or could potentially be used for measuring quality of cancer care in the population-based primary and acute or chronic setting across the life cycle of cancer-care continuum?”

### Identification of relevant studies

Electronic databases such as PubMed, Medline, PsycINFO, Cochrane Library, Ovid EMBASE, KISS, KoreaMed, and RISS were used to search and identify the relevant scientific literature from May 2020 to September 2020 using a predefined search strategy. The search focused on articles published between 2010 and 2020, and the search terms were cancer OR cancer patients OR survivors AND monitoring AND indicator AND early detection^*^ OR intervention^*^ OR treatment^*^ OR drug OR quality of life OR survivor^*^ OR end of life OR palliative care OR Review^*^ OR systematic review/ OR meta-analysis/ OR Guideline. These were reviewed to identify evidence-based recommendations.

The next step examined the existing national and international guidelines regarding the entire life cycle of cancer care based on the website of the American Society of Clinical Oncology and the guidelines of the European Union, World Health Organization, IARC, OECD, National Health Service, Canada, Japan, and South Korea. Some non-English guidelines were translated by the research team. Furthermore, problems resulting from unclear translation or unclear formulation were resolved through team discussion.

The next process was initiated by combining both the literature review and additional indicator guidelines. Each concept was converted into an indicator by formulating a definition, numerator, and denominator. All converted topics were checked for loss of information that could result from the research team's translation.

### Study selection

All search results were exported from the electronic databases in the Research Information Systems format and imported into EndNote. Titles and abstracts were screened for eligibility, and duplicates were removed. The screening process was repeated by three researchers to ensure reproducibility and verify the outcomes. Articles that were finally selected for a full-text review were uploaded into EndNote. The quality of the studies was assessed using the PRISMA checklist for cross-sectional studies. The research team (VR, JJ, JAK, and JHK) conducted the appraisal collaboratively. The reviewed articles were independently assessed by two reviewers (VR and JJ) for fit and relevance, and full-text versions were evaluated to determine inclusion and exclusion. Team reviewers (JS, JAK, JHK, and M.C) examined the reviewed articles to resolve any disagreements through several meetings. All the review-related discussions and regular meetings were conducted. All the team members were unable to attend the regular meeting in person due to the COVID-19 pandemic. As a result, a hybrid mode that included offline and online Zoom conversations was deployed to distinguish between literature search and data synthesis.

#### Inclusion criteria

This study focused on original articles on cancer indicators or index measurement activities and did not filter items based on country of origin, publication year, or publication status. Studies using mixed methods, including the Delphi technique, were also included. Studies on cancer indicators, early detection, quality of care, treatment, and end-of-life surveillance were prioritized for review.

#### Exclusion criteria

Studies published in other than English were excluded. In addition, poster presentations, commentaries, review articles, letters to the editor, and editorials were excluded. Articles that were not related to the quality of care and/or cancer indicators were excluded.

### Charting the data (data extraction)

Data were extracted in PRISMA-ScR Checklist (Supplementary Table S1) and organized the selected articles. Data collected from the literature were reviewed independently by the three researchers. Whenever discrepancies were identified, the researchers discussed these discrepancies and resolved them through consensus. The results were summarized in an Excel spreadsheet.

### Collating, summarizing, and reporting the results

**Figure 2:**
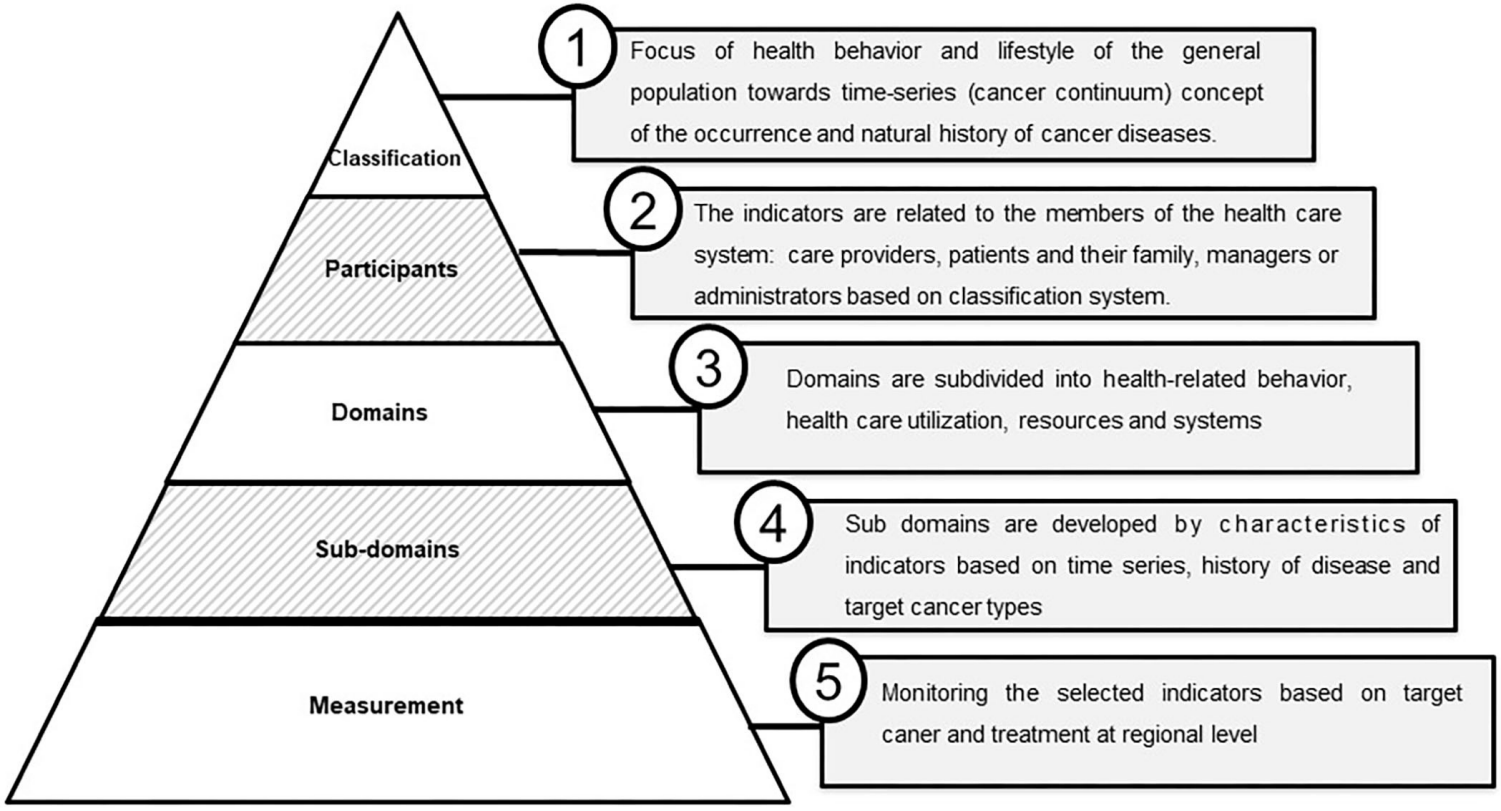
Vertical integration and significance cancer indicators monitoring by region. Indicators are composed of five vertical systems: “Classification,” “Participants,” “Domains,” “Subdomains,” and “Measurement.”

The extracted data chart is collated from the reviewed articles and summarized according to the study characteristics, including the author(s), publication year, country, purpose, research design, setting, target population, and methods of data collection, with the number of indicators and their characteristics (Supplementary Table S2). The findings were presented as a narrative synthesis and reported based on the heterogeneous nature of the reviewed articles. The selected indicators were composed of five aspects, namely “Classification,” “Participants,” “Domains,” “Subdomains,” and “Measurement” (Figure 2). The classification focused on the health behavior and lifestyle of the general population toward the time-series (cancer continuum) concept of the occurrence and natural history of cancer diseases. Participants are considered patients (patients with cancer, including survivors), providers (the physician or oncologist who is evaluating the cancer-related medical services and resources), and family members of the patient with cancer. Domains indicate the health-related behavior of the patient such as healthcare utilization, resources, and availability of facilities. Subdomains are derived from the characteristics of the indicators based on time series, history of disease, and target cancer types. Measurement denotes the monitoring or assessment of the indicators based on target cancer and treatment (Supplementary Tables S3, S4).

### Consultation

Consultations were held with experts on cancer indicators and monitoring (e.g., medical professionals, oncologists, academics, and researchers). A multidisciplinary expert's opinion included forty-two experts to select the cancer measures according to the priority. We divided the experts into three groups, namely, researchers, policymakers, and clinicians. Then, we randomly selected five researchers, three policymakers, and three clinicians. Of the five researchers, two were from the internal research group, and the remaining three were from the national cancer center and other colleges. Of the three policymakers, one was a director of a public or community health service center, another from the Ministry of Health and Welfare, and the other from the National Cancer Center. For clinicians, we selected one oncologist, one internal physician, and one radio-oncologist. All expert members were contacted *via* email; the purpose of the project was explained, and consent was obtained. They reviewed and suggested revisions to the thematic chart based on the cancer life cycle, such as prevention, early diagnosis, treatment, quality of care, survivor management, and end-of-life care, and pointed out gaps in the indicators and the connection to community assessment.

## Results

### Literature search and characteristics of the articles

**Figure 3:**
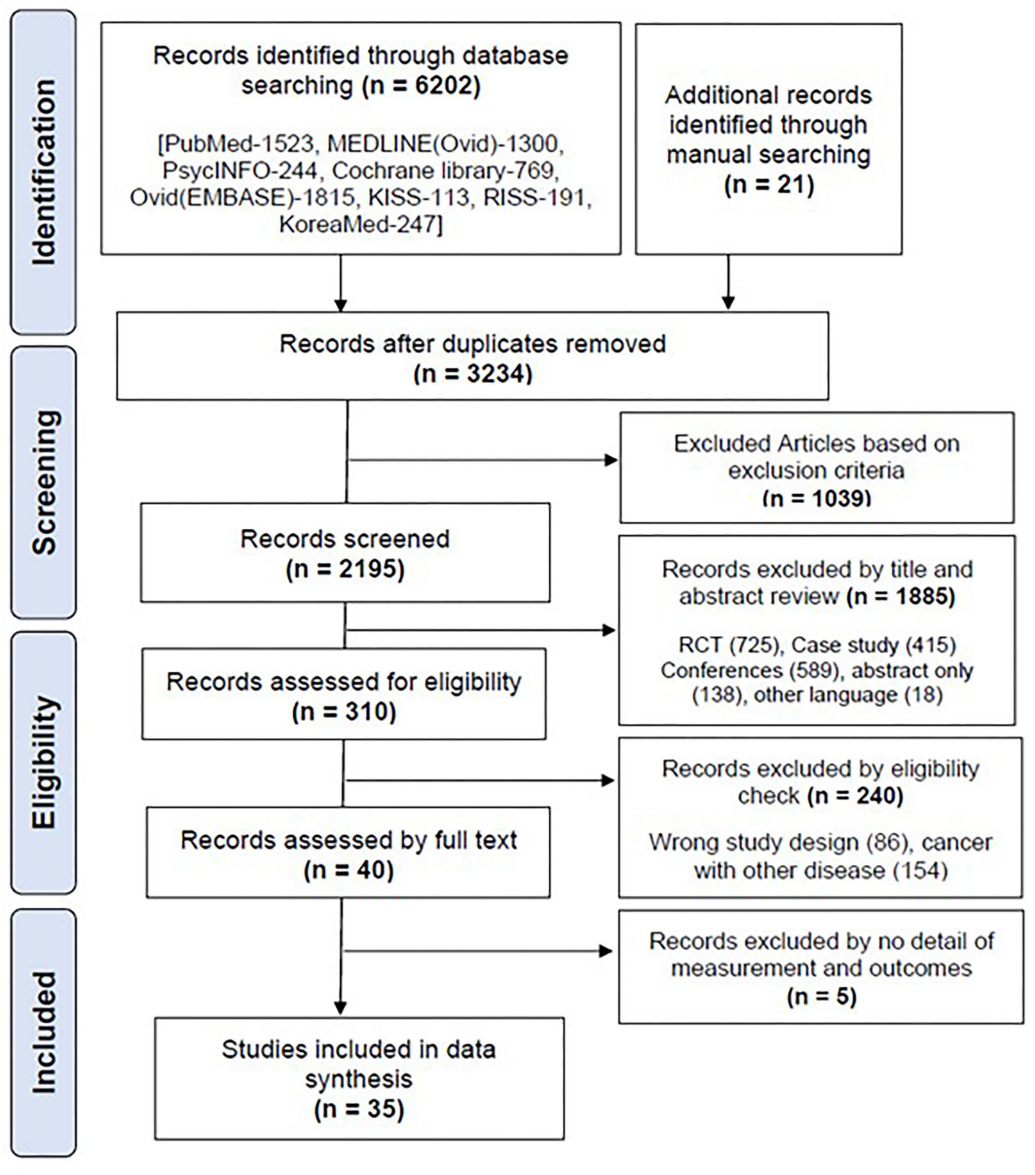
Flow diagram for the literature search and article selection process.

A total of 6,202 articles were retrieved from the database, and 21 articles were retrieved through a manual search. A total of 3,234 articles remained after the removal of duplicates, and 2,195 were selected for screening. The retrieved documents were screened for eligibility based on their titles and abstracts. Finally, 35 articles (11–45) were selected for the final data synthesis (Figure 3).

The characteristics of these studies are presented in Table 1. The number of most published articles included five studies in Korea (12, 20, 37, 38, 42) and the United States (11, 14, 21, 26, 32), three studies in the Netherlands (23, 25, 27) and multinationals (16, 29, 33), and two studies in Switzerland (13, 34), Australia (31, 41), Canada (19, 43), and Europe (17, 36). Only one least published study was included from the American Society of Cancer Organization (ASCO) (15), China (44), France (24), Italy (39), Morocco (28), Norway (35), Saudi Arabia (40), the UK (45), and Spain (18), and according to the year of publication, 75% of the articles were published between 2017 and 2020, indicating that the selected articles were relatively new with different details (Supplementary Figure S1). The number of studies on target cancers in selected articles included seven studies focused on all types of cancers (12, 20, 21, 36, 42, 43, 45), six studies on colorectal (13, 17, 25, 27, 32, 33), five studies on breast (23, 28, 35, 39, 44), three studies on cervical (16, 30, 40), lung (37, 38, 41), and bladder (11, 14, 19), two studies on each ovarian (24, 26) and prostate (29, 31), and one study on each colon (18), testicular (22), mesorectal (34), and palliative care (15) (Supplementary Figure S2). Most of the studies conducted in acute cancer medical and surgical settings in hospitals (11–13, 18, 23, 24, 27, 28, 30, 33–35, 37–39, 44). Some of the studies have been initiated by healthcare organizations and national-based projects by institutions. All the details of the study characteristics are described in Table 1.

**Table 1:** Description of the selected review articles.

**References**	**Country**	**Methods**				**Outcome**
		**Design**	**Target cancer**	**Setting**	**Data collection**	
Leow et al. (11)	USA	Systematic review	Bladder	Hospital	Urologic surgery and special procedure data	Urologic Surgery/Center for Surgery and Public Health
Min JK et al. (12)	Korea	Modified Delphi method Expert Panel	All	Hospital and public health center	National cancer screening data	Indicators update and Propose performance targets.
Bianchi et al. (13)	Switzerland	Literature review Delphi method	Colorectal	Hospitals	Ticino Cancer Registry	Set of quality indicators for whole diagnostic-treatment
Nazemi et al. (14)	USA	Cohort study	Bladder	National Cancer Institute's SEER	National cancer institute's surveillance, epidemiology data	Overall survival rate and Disease-specific survival rate
Carlos et al. (15)	ASCO	Retrospective study	Palliative care	Hospital, Clinic & Community center	Death records of palliative care center data	Perceived improvements in pain management
Partanen et al. (16)	Sweden Norway	Network analysis	Cervical	National health center	Population based screening data	Increased coverage rates and quality assurance
Altobelli et al. (17)	Europe	Randomized controlled trials	Colorectal	WHO	CRC screening based on stool testing	Guideline for resources available
Sancho-M et al. (18)	Spain	Prospective cohort study	Colon	Hospitals	Population based colon surgery data	Identify risk factors for anastomotic leakage after bowel resection
Khare et al. (19)	Canada	Modified Delphi method, Expert Panel	Bladder	Canadian Urologic Oncology Group	Scientific article	Development of quality indicators
Jung et al. (20)	Korea	Cohort study	All	National Cancer center	Korea National Cancer Incidence Database	Age-standardized rate of cancer continuum
Ramirez et al. (21)	USA	Mixed method	All	Community and clinic	CBPR data	Quality of life of both general and disease-specific treatment follow-up
Vlayen et al. (22)	Belgium	Systematic review Expert panel	Testicular	Belgian Health Care Knowledge Center	Belgian Cancer Registry data	Update- Evidence-based clinical practice guidelines
Vos EL et al. (23)	Netherland	Validation study	Breast	Hospitals	Nationwide, population-based registry	Variation of Quality of indicators according to hospital size
Gac et al. (24)	France	Retrospective cohort study	Ovarian	Cancer care hospitals	European commission quality indicators data	Evaluate the quality of ovarian cancer patient management
Keikes et al. (25)	Netherland	Systemic review	Colorectal	Academic Medical Center	Scientific article	Development, evaluation, and validation of quality indicators
ElNaggar et al. (26)	USA	Intervention study	Ovarian	Comprehensive Cancer Center	Institutional ovarian cancer database	Assess Society of Gynecologic Oncology quality measure
Gooiker et al. (27)	Netherland	Expert Panel	Colorectal	Hospital	Nationwide, web-based database	Validate the quality indicators
Charaka et al. (28)	Morocco	Retrospective study	Breast	Public Health Center Hospital	National screening test data	Monitoring quality improvement and enhance performance indicators
Sampurno et al. (29)	USA, UK Australia	Modified Delphi method Expert panel	Prostate	Guideline Development	Online survey and face-to-face meeting	Propose and update the quality indicators guidelines
Watanabe et al. (30)	Japan	Modified Delphi method	Cervical	Hospital	Hospital-based cancer registry Insurance claims data	Adherence of standards of care across Japan
Tsiamis et al. (31)	Australia	Literature Review Modified Delphi Method	Prostate	Radiology care center	Prostate Cancer Outcomes Registry-Australia & New Zealand	Develop quality indicators and monitor radiotherapy care
Boland et al. (32)	USA	Cohort study	Colorectal	National Cancer Institute	National Cancer Data Base	Stage-specific survival
Jackson et al. (33)	USA, UK Ireland Newzeland Australia	Modified Delphi method Expert panel	Colorectal	Hospital Cancer research center	NCCN and CCQMS Guidelines	Update long-term survival rate guideline
Schneider et al. (34)	Switzerland	Retrospective study	Meso rectal	Hospital	Medical records	Demonstrate the local reoccurrence rate and special treatment
Hartmann-Johnsen et al. (35)	Norway	Longitudinal study	Breast	Hospitals and clinic	All medical entries and Curate data	Estimation of the quality indicators for all hospital, treating breast cancer
Baili et al. (36)	Europe	Systemic review Expert panel	All	European cancer indicator project center	Population-based cancer registry database	Estimate the rehabilitation indicators in individual country and comparable across Europe
Yeo et al. (37)	Korea	Descriptive study	Lung	Tertiary and general hospitals	Health Insurance Review and Assessment	Implementation of newer indicators in lung cancer care
Lee et al. (38)	Korea	Literature review	Lung	Hospital	Scientific article	Estimation 3-years survival rate
Biganzoli et al. (39)	Italy	Literature Review Benchmarking	Breast	Hospitals Breast cancer centers	Scientific article	Set the international and national level to audit quality indicators
Anfinan and Sait (40)	Saudi Arabia	Retrospective study	Cervical	Tertiary care center	Medical records	8-years survival rate
Kim et al. (41)	Australia	Literature review Expert Panel	Lung	Regional center	Scientific article	Update and implement the existing guidelines
Choi et al. (42)	Korea	Retrospective study	All	GLOBOCAN and OECD countries	GLOBOCAN 2012 database for all 34 OECD countries	Development of national cancer control policies in cancer screening
Rae et al. (43)	Canada	Modified Delphi method Expert panel	All	Research center	Existing indicators of adolescent and young adult [AYA] guidelines	Update the system performance indicators
Wang et al. (44)	China	Retrospective study	Breast	Hospitals	Medical history record	Estimations of survival rate
Muller et al. (45)	UK	Gray literature	All	Research center	Scientific article and Data	Adherence to clinical quality indicator

USA, The United States of America; UK, United Kingdom; ASCO, American Society of Cancer Oncology; SEER, The Surveillance, Epidemiology, and End Results Program; WHO, World Health Organization; CBPR, Community, Based Participatory Research; NCCN, The National Comprehensive Cancer Network; CCQMS, Cancer Care Quality Measurement System; GLOBOCAN, Global Cancer Observatory; OECD, The Organization for Economic Co, operation and Development.

The national and international guidelines for cancer care search comprised 1,116 indicators (46–65), and all the details included organizations such as WHO (52), IARC (46), OECD (49), ASCO (50), National Health Service (51), the UK, Center for Cancer Control and Information Services, National Cancer Center, Japan (53–59), National Cancer Center (NCC), Korea (60), and other government and non-government organizations (61–65). All the details such as name of the organization, year of publication, population coverage, and number of indicators are listed in Table 2. All the reviewed articles and guidelines were converted into indicators that were linked to either prevention, early diagnosis, treatment, quality of care, survivor management, or end-of-life care.

**Table 2:** Characteristics of the national and international cancer guidelines.

**Level**	**Cancer guidelines and reports**	**Organization**	**Publication**	**Population coverage**	**No.of indicators**
International Guidelines	Cancer control and world cancer report (46)	International Agency for Research on Cancer [IARC]	2009–2019	International	127
	Canadian cancer society (47)	CANADA	2015–2019	National, Regional	122
	European guide for quality national cancer control programs (48)	Europe	2013–2019	National	101
	National cancer care plan (49)	The Organization for Economic Co–operation and Development [OECD]	2013–2019	International	150
	Quality oncology practice initiative (50)	American Society of Clinical Oncology [ASCO]	2013–2019	National	98
	Scottish cancer task force national cancer quality steering group guidelines and indicators (51)	National Health Service [NHS]	2013–2020	National	116
	WHO–Cancer care Report for ALL (52)	World Health Organization [WHO]	2005–2020	International	268
	National cancer registration hospitals such as cancer treatment–linked hospitals (53)	Center for Cancer Control and Information Services, National Cancer Center– Japan	2017	National	12
	Cancer hospital– five–year survival rate report 2010–2011 (54)		2019	National	12
	Cancer care linked hospital– Survival rate report – 2013 (55)		2019	Regional	7
	Cancer statistics 2019 (56)		2019	National	25
	National screening programme DATA book (57)		2020	National	7
	Monitoring of cancer incidence in Japan (58)		2019	National	15
	Cancer incidence rate in Japan (59)	Cancer and Disease Control Division, Ministry of Health, Labor and Welfare–Japan	2020	National	4
National Guidelines	Cancer statistics (60)	National Cancer Center [NCC] Korea	2007–2020	National	9
	Measures to improve the classification system and evaluation of health indicators (61)	Health Insurance Review and Assessment Service Korea	2019	Regional	1
	Regional health index (62)	Korea Health Ranking [Elio & company]	2018	Regional	1
	Regional health vulnerable indicators (63)	Korea Health Promotion Institute	2016	Regional	8
	Community health Survey–Health indicators (64)	Hallym University	2013	Regional	5
	Community health Survey (65)	Center for Disease Control Korea	2008–2019	Regional	28

### Characteristics of cancer indicators

A wide variety of 254 cancer indicators and measures are retrieved across the literature as a whole and listed in Supplementary Table S5. Consistent with its search strategies, the study found literature reviews and guidelines that combined population-based monitoring with the entire life cycle of cancer care. Over all findings of the cancer indicators according to the classification, most of them were treatment (33.5%), then primary prevention (24%), secondary prevention (18.1%), quality of life (13%), end-of-life care (6%), and less percentage found in survivor management (5.5%). In terms of participants, most of them were administrators (154/254), providers (73/254), and then patients (17/254) based on the classifications (Table 3). The subdomains are included specific procedures and treatments are widely used in cancer care settings (70/254), followed by hospital professionals (28/254) and incidence-related cancer indicators (18/254). All the frequency and percentage of participants and subdomains based on the main classifications are given in Tables 3, 4.

**Table 3:** Distribution of cancer indicators according to the participants.

**Classification/** **participants**	**Primary prevention**	**Secondary prevention**	**Treatment**	**Quality of care**	**Survivor management**	**End of life care**	**Total**
Patient	8	2	3	1	1	2	17
Provider	–	2	59	5	2	5	73
Administrator	49	39	20	27	11	8	154
Provider/administrator	2	2	1	–	–	–	5
Patient/administrator	2	–	–	–	–	–	2
Patient/family	–	1	–	–	–	–	1
Patient/family/administrator	–	–	1	–	–	–	1
Patient/administrator/provider	–	–	1	–	–	–	1
**Total (%)**	**61 (24.0)**	**46 (18.1)**	**85 (33.5)**	**33 (13.0)**	**14 (5.5)**	**15 (6.0)**	**254 (100)**

**Table 4:** Distribution of cancer indicators according to the sub domains.

**Classification/** **subdomains**	**Primary prevention**	**Secondary prevention**	**Treatment**	**Quality of care**	**Survivor management**	**End of life care**	**Total**
Smoking	14	–	–	–	–	–	14
Alcohol	14	–	–	–	–	–	14
Nutrition	3	–	–	–	–	–	3
Obesity	3	–	–	–	–	–	3
Physical activity	4	–	–	–	–	–	4
Research & investment	8	–	–	–	–	1	9
High risk for infection	3	1	–	–	–	–	4
High risk for chronic disease	1	–	–	–	–	–	1
High risk for occupation environment	4	–	–	–	–	–	4
Health care system	1	4	2	2	2	1	12
Health professionals	5	11	–	3	2	7	28
Vaccine/immunization	1	–	–	–	–	–	1
Incidence	–	3	–	–	–	–	3
Diagnosis	–	18	–	–	–	–	18
Prevalence	–	1	1	1	–	2	5
General health checkup	–	1	–	–	–	–	1
Facilities	–	2	–	–	–	–	2
Patient–centered	–	3	3	3	–	2	11
Treatment/consultation/ interruption rate	–	1	3	1	7	–	12
Treatment plan & record	–	–	16	–	1	–	17
Specific procedure and treatment	–	1	60	5	3	1	70
Health expenditure	–	–	–	5	–	–	5
Survival/mortality	–	–	–	13	–	–	13
**Total**	**61[24.0]**	**46[18.1]**	**85[33.5]**	**33[13.0]**	**15[5.9]**	**14[5.5]**	**254[100]**

## Discussion

The aim of this study was to review the scientific articles and international guidelines for the development of cancer indicators and measurement for monitoring the life cycle of cancer care. We have appraised the use and reporting of scoping reviews from electronic databases to select cancer indicators, and our review retrieved 35 scientific articles and national and international cancer care guidelines by gray literature search. Most of the studies from Korea and the USA in equal and consists of population- and patient-level indicators between 2010 and 2020. There was remarkable variability in terms of cancer indicators among selected studies. Our findings were synthesized and reported by

classifications, participants, domains, subdomains, and measurements. It has been suggested that the population-based monitoring of the entire life cycle of cancer care would assess primary prevention, secondary prevention (early detection and diagnosis), treatment, quality of care, survivor management, and end-of-life care. All the classifications are interconnected with the domains and subdomains. Moreover, the study reports of all the guidelines and reviewed contents were converted into cancer indicators and further classified.

Cancer prevention can be achieved through primary, secondary, and tertiary methods. Primary prevention involves the practice of healthy behaviors to lower one's risk of developing cancer (10, 47). This study found 61 indicators related to primary prevention that focused on the subdomains of obesity, physical activity, research and investment, alcohol, smoking, nutrition, high risk of infection, high risk of chronic disease, high risk of occupation and environment, medical care system, health professionals, and vaccination-immunizations. The Center for Disease Control recommends a healthy diet and at least 30 min of moderate physical activity on 5 or more days every week or at least 20 min of vigorous activity on 3 or more days every week (66). Some studies have addressed the empowerment and education of people to facilitate healthy lifestyle choices related to tobacco use (14, 46) and nutrition (46, 48, 51, 58). The IARC reported a positive relationship between obesity and the incidence of several types of cancer, including postmenopausal breast cancer and cancers of the colon, endometrium, esophagus, and kidney. Some studies have also addressed the promotion and provision of vaccines that prevent cancer, such as the human papillomavirus (HPV) vaccine (49, 51, 52, 63, 67).

A total of 46 indicators related to secondary prevention were identified, such as those concerning early detection and diagnosis (59, 63, 65), incidence and prevalence rates (59–61), healthcare system (49, 51, 52), and healthcare professionals (15, 16, 32–34, 37, 46, 53). Patient-oriented care and other cancer-related consultations were included in this second group of cancer indicators. Cancer screening in the general population refers to detecting cancer when no apparent symptoms are present. This is done with the aim of decreasing cancer-related morbidity and mortality (12, 52, 59–61). Indicators related to cancer screening were proposed and reinforced by several articles and guidelines (24, 26, 28, 32, 37, 47, 50, 53, 58, 60, 65). Cancer screening has been reported to be effective, and screening tests must meet two criteria. First, the screening test should detect cancer prior to the development of symptoms. Second, the treatment should be initiated as soon as the presence of cancer is confirmed, which would result in improved outcomes (16, 17, 28, 47, 48, 64, 67).

A total of 85 indicators related to treatment and appropriate special procedures were identified. Most treatment-related indicators are handled by health professionals who provide specialized care and monitoring (15, 16, 32, 48, 55). Several studies have reported appropriate treatments or procedures, such as an endoscopy for stomach cancer (14), mammography for breast cancer (13, 17, 23, 28, 35, 39, 42, 52), computed tomography (CT) and positron emission tomography (PET) for malignant cancer at each stage (37, 48, 50), sedated auditory brainstem response (SABR) for lung cancer (37, 38, 41, 51), molecular test (13, 16), colonoscopy for colorectal cancer (32, 38, 39, 43, 44, 48), and colposcopy (14, 16, 18, 32, 49). In addition, international guidelines have discussed the treatment regimen of medications (16, 48, 51, 53, 54, 62), chemotherapy, radiotherapy, and neoadjuvant therapy (13, 34, 35, 48, 51, 53–58). Surgical indicators, such as granulocyte colony-stimulating factor (17, 18, 50) and total or radical resection for stomach cancer and colorectal cancer (13, 17, 25, 27), were also derived. However, indicators related to the treatment of cancer must focus on its early stages to avoid the metastatic or chronic suffering of patients with cancer. All the discussed indicators to be used near death might be based on patient's preferences. Therefore, treatment-related indicators and their measurement are necessary to reduce morbidity or mortality rates. However, the measurement of treatment indicators depends on the availability of the quality data obtained without any burden and gap in order to predict the actual results. Especially, chemotherapy use in the chronic stage might be challenging to measure in the settings, frequency of episodes may not be tracked, which may affect the outcomes. Therefore, some of the treatment indicators may disagree regarding whether it represents low or poor quality of care or improper documentation.

A total of 33 quality of care indicators were identified, which focused on the care of patients with cancer and their quality of life. Most of the indicators involved healthcare managers or administrators, followed by providers and patients and their families. The selected indicators were medical expenditure (15, 17, 22, 46, 51, 61) after surgical complications and follow-up care (16, 21, 26, 48), possibility of the recurrence of cancer (15), medication follow-up (15, 16, 22, 46), and mortality rate after surgery or potential cases (49–52, 56–59, 62, 63). Studies have focused on the quality-of-care indicators for each type of cancer (21–32, 39, 41, 44, 47–49). This scoping review demonstrated the most important indicators related to the quality of care. Therefore, before measuring the quality of care, we must identify, develop, analyze, and validate the reliability of indicators that can be measured and compared to assess the improvement of cancer care.

A total of 15 survivor management-related indicators were identified. The participants were healthcare managers or administrators who are involved in the improvement of the quality of post-treatment patient care. Most of the patient-focused indicators were centered on family, such as psychosocial support services (14, 33, 36, 50), rehabilitation services (36, 46, 48, 50), alternative diversional activities (14, 57, 62, 64, 65), and follow-up care *via* hospital visits and health practice (46, 49, 51, 52, 56), and assessment and management of psychosocial pain (14, 33, 36, 50). However, there are many challenges in developing indicators for cancer survivor care from the national public health perspective. There is a need to develop structured training programs for survivor management alone to improve the quality of cancer care.

A total of 14 indicators related to end-of-life care were identified. Most of the participants were healthcare managers or administrators who are involved with the organizational structures, care facility centers, and patients. The subdomains retrieved were the prevalence of patients with cancer enrolled in the last year of palliative care (46, 47, 49, 52, 54) or hospice care (17, 18, 53). Health services and professionals are involved in quality improvement (35, 43–45, 51), recruitment of care providers and field workers (49, 50, 52, 55, 56), financial support for childhood cancer care (15, 60), number of patients receiving hospice care per year (46, 48, 50, 52), self-satisfaction survey of palliative care services (16, 38, 48, 49), and evidence-based research and investment (17, 18, 46–48) related to end-of-life care. It was found that some contrast of practical barriers exists to implementing end-of-life cancer indicators, identifying that the relevant population is challenging to measure the settings or databases due to the difficulties in predicting the end-of-life period. In addition, the indicators are based on precise document restrictions such as communication, patient-reported outcomes, or preferences.

To measure public accountability at the regional level, this scoping review measured the collected indicators using regional-level healthcare data, national cancer center data, national cohort, and health insurance claims data, as well as cancer-specialized hospital data. Furthermore, most of the quality indicators were measured by the structure, process, and outcome method of Donabedian's model for quality of cancer care (68). Therefore, selected cancer indicators will be designed to measure the entire life cycle of cancer care.

To discover the numerous gaps in the clinical care that is currently being delivered, additional implications of these findings will be applied among patients with cancer in community and clinical settings. We anticipate that our population-based measurements and preliminary cancer indicators will be useful directly or indirectly to other nations or areas looking to raise the standard of cancer care in accordance with their organizational structure.

### Limitations

There are some limitations in the existing studies and guidelines of cancer indicators from published scientific articles. First, it is possible that we did not find all the articles in this review, due to variance in keywords or search terms. Second, the presented conceptual framework for the entire life cycle of cancer care constructed by the research team, thus, would be needed to revise by the experts in future study. Third, there is unavailability of population-based indicators from individual studies; therefore, we could not be able to find the different methods of measurement of cancer indicators. Finally, we were not able to access the original data from the articles and international guidelines. Therefore, we would not be able to answer some questions including diagnostic procedures and treatment types. However, it is true that population-based monitoring of cancer indicators is a positive approach for measuring the entire life cycle of cancer care, and further extension of review would need to be conducted again in detail.

### Future directions

Further validation and reduction of the indicators are needed to develop cancer indicators for the entire life cycle of cancer care relevant to prevention, early diagnosis, treatment, quality of care, survivor management, and end-of-life care. Extended measures also need to be broader. Therefore, the next step is to conduct a modified Delphi and expert panel discussion to develop national cancer indicators for the entire life cycle of cancer care. However, additional significant cancer indicators might be available that can be tailored to suit the health system's goals and improve the quality of cancer care in Korea.

## Conclusion

The scoping review process provides guidance by selecting a subset of indicators that are widely accepted as relevant by those who will drive the improvement of the population-based monitoring of cancer indicators. As an additional advantage compared to the usual way of setting up national databases in South Korea, the present selected indicators will be revised by oncological professionals and the national cancer center committee. At present, there is a substantial interest in the development of cancer indicators in this field. The reviewed cancer indicators will be undergoing the Delphi process to further develop the current project. Thus, both the process and the resultant indicators may prove of interest to policymakers, clinicians, and researchers across South Korea.

## Data availability statement

The original contributions presented in the study are included in the article/Supplementary material, further inquiries can be directed to the corresponding author/s.

## Ethics statement

Ethical review and approval was not required for the study on human participants in accordance with the local legislation and institutional requirements. Written informed consent for participation was not required for this study in accordance with the national legislation and the institutional requirements.

## Author contributions

JS and MC conceptualized and supervised the project. JAK, JHK, VR, and JJ performed data curation and synthesis. VR and JS wrote the paper. All authors reviewed the manuscript. All authors contributed to the article and approved the submitted version.

## Funding

This scoping review was conducted as part of the Regional Cancer Center and National Cancer Center's Collaborative Cancer Conquest Promotion R&D Project, entitled Development of monitoring cancer indicators and measurement by statistical analysis (Project Number: HA20C0004).

## Conflict of interest

The authors declare that the research was conducted in the absence of any commercial or financial relationships that could be construed as a potential conflict of interest.

## Publisher's note

All claims expressed in this article are solely those of the authors and do not necessarily represent those of their affiliated organizations, or those of the publisher, the editors and the reviewers. Any product that may be evaluated in this article, or claim that may be made by its manufacturer, is not guaranteed or endorsed by the publisher.
