# Supplementary material for: A scoping review on population-centered indicators for cancer care continuum
Source: Front Public Health. 2022 Oct 14;10:912946. doi: 10.3389/fpubh.2022.912946 (PMC9614426; doi:10.3389/fpubh.2022.912946)
Supplement: Supplementary file 1 [file Data_Sheet_1.PDF]

Supplementary Materials

Supplementary Table 1. Preferred Reporting Items for Systematic reviews and Meta-Analyses extension for Scoping Reviews (PRISMA-ScR) Checklist

SECTION	ITEM	PRISMA-ScR CHECKLIST ITEM	REPORTED ON PAGE #
Title	1	Identify the report as a scoping review.	1
Structured summary	2	Provide a structured summary that includes (as applicable): background, objectives, eligibility criteria, sources of evidence, charting methods, results, and conclusions that relate to the review questions and objectives.	2
Rationale	3	Describe the rationale for the review in the context of what is already known. Explain why the review questions/objectives lend themselves to a scoping review approach.	3
Objectives	4	Provide an explicit statement of the questions and objectives being addressed with reference to their key elements (e.g., population or participants, concepts, and context) or other relevant key elements used to conceptualize the review questions and/or objectives.	4
Protocol and registration	5	Indicate whether a review protocol exists; state if and where it can be accessed (e.g., a Web address); and if available, provide registration information, including the registration number.	NA
Eligibility criteria	6	Specify characteristics of the sources of evidence used as eligibility criteria (e.g., years considered, language, and publication status), and provide a rationale.	4-5
Information sources*	7	Describe all information sources in the search (e.g., databases with dates of coverage and contact with authors to identify additional sources), as well as the date the most recent search was executed.	4-5
Search	8	Present the full electronic search strategy for at least 1 database, including any limits used, such that it could be repeated.	5
Selection of sources of evidence†	9	State the process for selecting sources of evidence (i.e., screening and eligibility) included in the scoping review.	5
Data charting process‡	10	Describe the methods of charting data from the included sources of evidence (e.g., calibrated forms or forms that have been tested by the team before their use, and whether data charting was done independently or in duplicate) and any processes for obtaining and confirming data from investigators.	5
Data items	11	List and define all variables for which data were sought and any assumptions and simplifications made.	6
Critical appraisal of individual sources of evidence§	12	If done, provide a rationale for conducting a critical appraisal of included sources of evidence; describe the methods used and how this information was used in any data synthesis (if appropriate).	6
Synthesis of results	13	Describe the methods of handling and summarizing the data that were charted.	6

SECTION	ITEM	PRISMA-ScR CHECKLIST ITEM	REPORTED ON PAGE #
Selection of sources of evidence	14	Give numbers of sources of evidence screened, assessed for eligibility, and included in the review, with reasons for exclusions at each stage, ideally using a flow diagram.	6-7
Characteristics of sources of evidence	15	For each source of evidence, present characteristics for which data were charted and provide the citations.	6-7
Critical appraisal within sources of evidence	16	If done, present data on critical appraisal of included sources of evidence (see item 12).	7
Results of individual sources of evidence	17	For each included source of evidence, present the relevant data that were charted that relate to the review questions and objectives.	7
Synthesis of results	18	Summarize and/or present the charting results as they relate to the review questions and objectives.	8
Summary of evidence	19	Summarize the main results (including an overview of concepts, themes, and types of evidence available), link to the review questions and objectives, and consider the relevance to key groups.	8-9
Limitations	20	Discuss the limitations of the scoping review process.	9
Conclusions	21	Provide a general interpretation of the results with respect to the review questions and objectives, as well as potential implications and/or next steps.	10
Funding	22	Describe sources of funding for the included sources of evidence, as well as sources of funding for the scoping review. Describe the role of the funders of the scoping review.	10

JBI = Joanna Briggs Institute; PRISMA-ScR = Preferred Reporting Items for Systematic reviews and Meta-Analyses extension for Scoping Reviews.
* Where *sources of evidence* (see second footnote) are compiled from, such as bibliographic databases, social media platforms, and Web sites.
† A more inclusive/heterogeneous term used to account for the different types of evidence or data sources (e.g., quantitative and/or qualitative research, expert opinion, and policy documents) that may be eligible in a scoping review as opposed to only studies. This is not to be confused with *information sources* (see first footnote).
‡ The frameworks by Arksey and O'Malley (6) and Levac and colleagues (7) and the JBI guidance (4, 5) refer to the process of data extraction in a scoping review as data charting.
§ The process of systematically examining research evidence to assess its validity, results, and relevance before using it to inform a decision. This term is used for items 12 and 19 instead of "risk of bias" (which is more applicable to systematic reviews of interventions) to include and acknowledge the various sources of evidence that may be used in a scoping review (e.g., quantitative and/or qualitative research, expert opinion, and policy document).

From: Tricco AC, Lillie E, Zarin W, O'Brien KK, Colquhoun H, Levac D, et al. PRISMA Extension for Scoping Reviews (PRISMAScR): Checklist and Explanation. Ann Intern Med. 2018;169:467–473. doi: 10.7326/M18-0850.

Supplementary table 2. Data extraction form for selected articles

Study ID	Authors	Year	Country	Aims of the study	Methods					Analysis	Outcomes	What are the type of cancer indicators?	Strength and Limitations	Funding (Agency or Research or organization)
					Design	Target cancer	Target population	Setting	Data collection/ Measurement					

Supplementary Figures

Supp. Figure 1

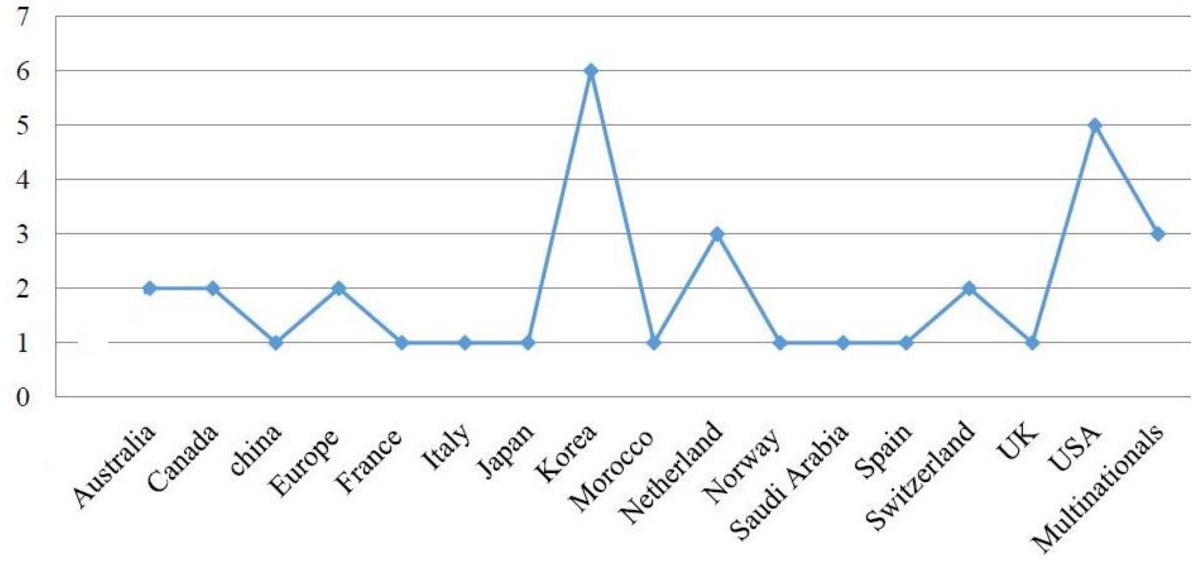

Supp. Figure 1. Number of articles published in countrywide.

UK, United Kingdom; USA, The United States if America

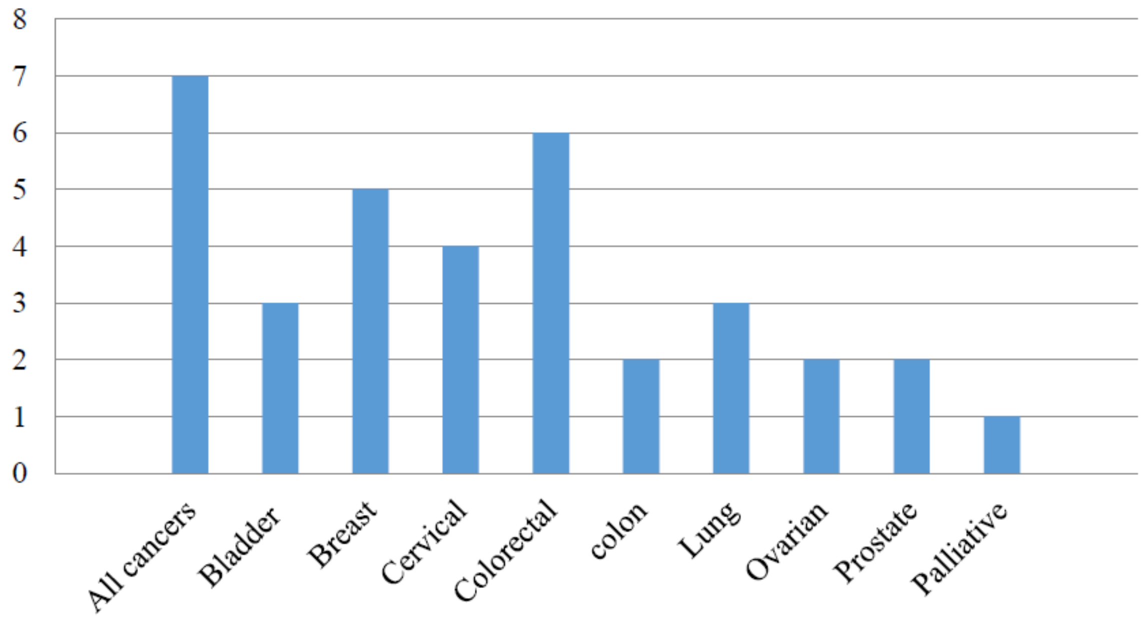

Supp. Figure 2. Number of target cancers in reviewed the articles.

Supplementary Table 3. Specification of participants involved in monitoring cancer indicators

Participants	Content
Patient	- The general population (potential cancer patients) including the people undergoing cancer treatment and survivors.<br>- Indicators related to patient-centered medical services or health care delivery systems of cancer.
Provider (Cancer care)	- It indicates cancer care professional, who is evaluating the cancer related medical services and resources.
Administrator/ organizer	- Evaluation indicators for policy maker such as cancer related medical service facilities, public health relations and patient support system.

Table 1 describes the specification of participants involved in population-based monitoring cancer care and measurement. It denotes patient – Individual and family involving cancer care; Provider- Physician, Oncologists, and Radiologist; Organizer- Managers, Healthcare workers or public relation officers in cancer care.

Supplementary Table 4. Specification of the Sub-domains in monitoring cancer indicators

Sub-domains	Content
Obesity	- Indicators related to anthropometric variables measurement of height (cm), weight (kg), BMI (kg/m2)
Smoking	- Indicators related to tobacco consumption and smoking cessation behavior
Alcohol	- Indicators related to alcohol consumption and behavior
Nutrition	- Indicators of nutritional behavior related to cancer
Physical activity	- Indicators of cancer related physical activity (e.g., survivor)
Research investment	- Indicators related to research investment for improvement of medical knowledge and system improvement
High risk for infection	- Indicators related to cancer prevention and related health behavior due to infection
Risk for chronic disease and occupational environment	- (Chronic diseases) Current status and related indicators of cancer-related chronic diseases<br>- (Occupational environment) Indicators related to exposure risk and monitoring of occupational cancer diseases
Health care system	- Cancer-related medical services and related policy indicators<br>- Cancer prevention, medical delivery system and related policy indicators
Healthcare professions	- General medical supply level and related indicators in the treatment area<br>- Prevention, diagnosis and treatment of cancer and related medical supply level and related indicators
Vaccine/immunization	- Other indicators related to cancer prevention (e.g., HPV)
Incidence	- Cancer incidence and related indicators
Diagnosis	- Indicators related quality of screening and national programme
Prevalence	- Cancer prevalence and related indicators<br>- Indicators related to registration process in medical services by patients
General health checkup	- Indicators of general health checkup that focus the potential cancer or manage risk factors related health behavior
Facility	- Facility indicators, that provide the information of early diagnosis, treatment, and aftercare of cancer care
Patient-centered care	- Indices related to customized treatment and monitoring according to the patient's characteristics<br>- Indicators related to emotional and social support of patients and their families
Consultation/treatment/ interruption rate	- Indicators related to patients with appropriate cancer treatment, treatment, and discontinuation of cancer treatment
Treatment plan and report	- Indicators related to medical records for completeness of treatment<br>- Indicators related to the evaluation of whether to establish evidence-based tr eatment plans during treatment
Appropriate procedure and treatment	- Indicators related to the adequacy of cancer treatment and monitoring plans and progression for curative purposes
Health expenditure	- Cancer related medical services and related indicators<br>- Cancer treatment and related medical expenses support system and related indicators
Surveillance/Mortality	- Indicators related to survival management and death

Supplementary tale 5. Description and list of 254 cancer indicators for population-based cancer indicators monitoring and measurement

Sl. No	Classification	Participants	Subdomains	Indicators	Measurement	Reference
1	Primary prevention	Administrator	Obesity	Obesity population rate	Population based MI distribution, appropriate weight ratio (BMI), gender, child obesity rate	47, 49, 48, 64, 65
2		Administrator	Obesity	Annual weight control attempt rate		52
3		Administrator	Obesity	The risk of obesity contributing to cancer	Age and Gender	47
4		Patient	Smoking	Reasons for using e-cigarettes	By e-cigarette type (cigarette type, liquid type)	63
5		Patient	Smoking	Exposure rate of Secondhand smoking	In the home, in the workplace, in public places	46, 47
6		Patient	Smoking	Number of treatment attempts for tobacco dependence	Medical records, by age, sex and yearly	48, 63
7		Administrator	Smoking	Smoking rate and smoking amount (per person)	Daily, year, gender, region, chronic disease, age (10-14 years old, over 15 years old, adults)	46
8		Administrator	Smoking	Current usage rate of e-cigarettes	By e-cigarette type (cigarette type, liquid type)	63
9		Administrator	Smoking	Exposure rate of secondhand smoke among non-smokers	Home environment, workplace, common places	47
10		Administrator	Smoking	The risk that smoking contributes to cancer	By age, gender	48, 63
11		Administrator	Smoking	Current smoker's - smoking rate within one month	Self-survey report (National Health data)	49
12		Administrator	Smoking	Smoking attempt rate of current smokers	In the home, in the workplace, in public places	46, 47, 49, 49, 59, 65
13		Administrator	Smoking	Recommended rate of smoking management in public places	Recommendation: Ethical standards and practices for smoking prevention	48, 63
14		Administrator	Smoking	Whether smoking cessation education program is in operation	By educational institution, primary medical environment, workplace, and country	49
15		Administrator	Smoking	Annual smoking education experience rate	Self-survey report (National Health data)	47
16		Administrator	Smoking	Public Health Center Smoking Posters and Smoking Pamphlets Usage Rate	Number of programme population covered (Distribution of posters and pamphlets)	32, 65

Sl. No	Classification	Participants	Subdomains	Indicators	Measurement	Reference
17	Primary prevention	Patient/Admin	Smoking	Cigarette sales	By yearly	47
18		Patient	Alcohol	Alcohol consumption rate	According to time period (daily, weekly, annual), per capita, by gender, by population	49, 48
19		Patient	Alcohol	Number of cancer patients due to alcohol intake	By cancer type (liver cancer, stomach cancer)	52
20		Patient	Alcohol	Number of admission and readmissions related to Alcoholism	readmission within 30 days/100,000 population, Use of medical records	49
21		Patient	Alcohol	Number of treatment attempts for alcoholism	Medical history of alcohol dependent patients	52
22		Administrator	Alcohol	High risk of alcohol intake rate	Medical records -Yearly, monthly, by target (drinking, alcoholic)	57, 63, 65
23		Administrator	Alcohol	Annual involuntary Commitment rate of Alcoholism	By region	63
24		Administrator	Alcohol	Annual involuntary Commitment rate of Alcoholism	By age	52
25		Administrator	Alcohol	Yearly alcohol abuse experience rate	National health survey data (self-evaluation data)	63
26		Administrator	Alcohol	Enforcement of alcohol prevention advertisement and regulations	Enforcement of alcohol complications and advertising regulations for children/adolescents (Educational institutions, public health center)	47
27		Administrator	Alcohol	Lifelong experience rate of Alcohol awareness education	National health survey data (self-evaluation data)	63
28		Administrator	Alcohol	Annual alcohol consumption reduction rate	National health survey data (self-evaluation data)	63
29		Administrator	Alcohol	Annual drunken driving experience rate	National health survey data (self-evaluation data)	63
30		Administrator	Alcohol	Risk of Contribution to Cancer of Drinking	By age, gender	46
31		Patient/Admin	Alcohol	Annual sales rate of alcoholic beverages	By population group, by country	47

Sl. No	Classification	Participants	Subdomains	Indicators	Measurement	Reference
32	Primary prevention	Administrator	Nutrition	Nutrient intake rate	Daily calorie intake calculationn [Fat, fruit and vegetable intake, calories, by population group (adult, adolescent)]	46, 48, 51, 58, 59
33		Administrator	Nutrition	Regular eating habit rate	National health self-survey data	63
34		Administrator	Nutrition	Low-salt diet practice rate	Food habit and calorie intake (National health self-survey data)	63
35		Patient	Physical activity	Physical activity attitude	Daily life activity	49, 52, 62
36		Administrator	Physical activity	Physical activity practice rate	Survey for Intensity of regular activity (violent 3 days or more a week, moderate 5 days a week or more)	49, 52, 64
37		Administrator	Physical activity	Awareness rate walking course	Survey or interview questionnaire Participation rate of annual sports activity and community exercise program	60
38		Administrator	Physical activity	Reasons for non-participation in sports activities	Self-survey data	60
39		Administrator	Research &investment	Financial/research fund support for cancer research and development	Existence of investment in cancer epidemiology and public health research, per capita spending on cancer research	46, 53
40		Administrator	Research &investment	National cancer related joint research support or incentivize	National cancer guidelines review	46, 49
41		Administrator	Research &investment	Development or Utilization of clinical guidelines	Year and Region	47
42		Administrator	Research &investment	Availability of resources (Manpower, financial and technical) allocated to support research activities (data collection and quality assurance of cancer registration.)	by Regional and general hospitals	52
43		Administrator	Research &investment	Cancer Research Performance Status (International/National/Regional Cancer Center)	By cancer type, classification (diagnosis, treatment, prevention), Information sharing among stakeholders (health policy developer, program manager, researcher)	47, 54
44		Administrator	Research &investment	Availability of Shared Research related information	By cancer type and national	46, 48
45		Administrator	Research &investment	Improvement of cancer research/treatment functions in major cancer treatment facilities	By cancer type and research institute	46, 52
46		Administrator	Research &investment	Patient participation rate in clinical research	Consent form from patient based on target cancer	49

Sl. No	Classification	Participants	Subdomains	Indicators	Measurement	Reference
47	Primary prevention	Administrator	Preventive measure	Improvement of Breastfeeding practice rate (Prevention of Breast cancer)	by region, female population of primi mothers	49, 23
48		Administrator	Risk of infection	Helicobacter infection rate	By region	46, 53
49		Administrator	Risk of Infection	90% and more of vaccination for risk population against communicable disease	By vaccine (HPV, HBV, HIV), by population group (youth, women)	49
50		Administrator	Risk of Infection	Preventable Cancer-Related Vaccination Rate	Coverage of HPV, by population group (age, region)	49
51		Administrator	Risk of Chronic disease	Current status of cancer-related chronic diseases	Medical records (High blood pressure, diabetes, chronic disease rate, hepatitis, By region, asbestos)	52
52		Administrator	Risk of occupation and environment	Exposure rate of carcinogens by occupational environment	History of Radiation exposure and evaluation of safety measures	51, 53
53		Administrator	Risk of occupation and environment	Existence of occupational safety and health laws to avoid exposure to carcinogens		46, 48
54		Administrator	Risk of occupation and environment	Identification rate and monitoring rate of workers exposed to occupational carcinogens	Workplace, national labour welfare data	49, 52
55		Administrator	Risk of occupation and environment	Whether to implement an education program to prevent exposure to carcinogens in the occupational environment	By target persons (employees, public officials)	49
56		Administrator	Health Care system	The existence of a cancer prevention working group for implementation in the health system and other fields	Enforcement of cancer related awareness programme and regional based coverage (Educational institutions, public health center)	52
57		Administrator	General health checkup	Establishment and standardization of medical information system	Regular data quality assurance for medical information/cancer registration data quality and international comparability (complying with IARC and ENCR guidelines) - National Health data	48
58		Administrator	Health professionals	Number of general hospitals per population	Per 100,000 people, by region	52
59		Provider/Admin	Health professionals	Number of medical staff training institutions	By region	49
60		Provider/Admin	Health professionals	Distribution of medical staff to underprivileged areas	Per population (per 1,000 people, per 100,000 people), by workforce type, by year	52
61		Administrator	Health professionals	Current Status of health care personnel	By regional cancer care hospital	46, 47 49, 53, 53

Sl. No	Classification	Participants	Subdomains	Indicators	Measurement	Reference
62	Secondary prevention	Administrator	Facilities	Ratio by visit route	Availability of facility to reach cancer care center (Ambulance, Transport or volunteer services)	47
63		Administrator	Facilities	Power ratio from other facilities in the visit route	By facility	52
64		Patient	Incidence	Cancer incidence rate	By sex, age, region, per 10,000 (100,000) population, by all carcinoma/carcinoma, By population, and socioeconomic level, by symptom, by age standardized incidence rate and incidence rate	46, 47, 49, 48, 20, 42
65		Patient/Family	Incidence	Lifetime risk of cancer	By gender, age, population, genetic cause survey, region (county and territorial risk survey), lifestyle and habit survey, self-reported	52
66		Administrator	Incidence	The number of cancer predictions	Regular evaluation by gender, age, per 100,000 population, year, region, and country	49, 57
67		Patient	Early detection and diagnosis	Precision inspection rate (%)<br>Proportion of those require a close screening test, among them how many are undergone screening test	Close-up examination rate (%)	46, 49, 48, 52, 65
68		Provider	Early detection and diagnosis	Conducted or not conducted according to the guidelines, checklist implementation rate	National city, ward, village, prefecture	52
69		Administrator	Early detection and diagnosis	Cancer screening acceptance rate	Sex, age (35-65), cancer type, duration, population group, region, within 30 months 70% or more of the examination	63
70		Administrator	Early detection and diagnosis	Examination accuracy	Sensitivity, specificity	52
71		Administrator	Early detection and diagnosis	Reasons for non-examination of cancer screening	Cancer screening data, by region	49
72		Administrator	Early detection and diagnosis	Evaluation rate of cancer examination institutions	By institutions, region and hospitals	47
73		Administrator	Early detection and diagnosis	Satisfaction with national cancer early screening	patient satisfaction records by hospitals, clinics and public health center	47
74		Administrator	Early detection and diagnosis	Rate of special examination (endoscopy, MRI, pathology, histology, ultrasound) among cancer patients	By gender, age, cancer type, within 6 months (before starting tea)	52
75		Administrator	Early detection and diagnosis	Percentage of invasive cancers that did not involve lymph nodes per 1000 screenings	By cancer type (stomach, liver, colon, cervical cancer)	56

Sl. No	Classification	Participants	Subdomains	Indicators	Measurement	Reference
76	Secondary prevention	Administrator	Early detection and diagnosis	Invasive tumor size at screening (tumor size ≤ 15 mm)	By age (35-50 years old), sex, and cancer type	56
77		Administrator	Early detection and diagnosis	Early screening for childhood cancer	Per 1 million people, by gender, age, and cancer type (child cancer)	56
78		Administrator	Early detection and diagnosis	Cancer detection rate (%)<br>Percentage of people with cancer among screening examinees"	Cancer detection rate (%), by prefecture	56
79		Administrator	Early detection and diagnosis	Positive prediction among the subjects of the detailed examination (%)<br>The proportion of people who have found cancer among those who are judged to require close examination	Positive reaction accuracy rate (%), by region	56
80		Administrator	Early detection and diagnosis	Results of the examination program (sensitivity, specificity)	Governance outcome indicators (death rate, quality of life, complications, incidence, etc. can be viewed)	52
81		Administrator	Early detection and diagnosis	Additional video capture rate at the time of examination	Medical records	45
82		Administrator	Early detection and diagnosis	Cost-effectiveness	Cost-effectiveness of early cancer screening	52
83		Administrator	Early detection and diagnosis	Cancer stage at screening	Comprehensive, pre-treatment, by site, excluding and including intraepithelial cancer	53, 60
84		Administrator	Early detection and diagnosis	Ratio of cancer patients who receive regular cancer screenings to cancer patients every year	By yearly, regional based survey	52
85		Administrator	Risk of infection	Ratio of the number of cancer cases due to infection to the total number of cancer cases	By infection (H. pylori, Opisthorchis viverrini, Clonorchis sinensis, and Schistosoma haematobium, /HBV and HCV, HHV, HTLV)	46
86		Administrator	Prevalence	Cancer prevalence	By region, hospital type, number of registered cases, cancer stage, age-adjusted morbidity rate (per 100,000 population), crude morbidity rate, cumulative morbidity rate, standardized morbidity	59
87		Administrator	Health Care system	Whether to invest for cancer screening	Review the cancer care policy	52
88		Administrator	Health Care system	Time taken from examination execution to examination result (positive/negative) information	Cancer type	49, 15
89		Administrator	Health Care system	Time taken from occurrence of suspicious symptoms to diagnosis	By cancer type, the time it took for the first checkup to be negative but positive for the second diagnosis test, and the time for the first and second checkups to be negative for symptoms but to be positive for the third diagnosis	49

Sl. No	Classification	Participants	Subdomains	Indicators	Measurement	Reference
90	Secondary pre vention	Administrator	Health Care system	Whether to establish a medical delivery system for access to treatment and economic access	Community Health Center	52
91		Administrator	Health professionals	Number of psychosocial experts for cancer management treatment	Per population (per 1,000 people, per 100,000 people), by year, by gender	47, 48
92		Administrator	Health professionals	Number of oncologists, number of residents	Country, year, territory, gender	32, 33, 47,
93		Administrator	Health professionals	Number of oncologists, number of residents	Per 100,000, by region, hospital, or clinic	46, 53
94		Administrator	Health professionals	Available facility to be count in cancer care canters	Total cancer, by cancer type (stomach, colon, colon, rectum, liver, lung, female breast, esophagus, pancreas, cervix, uterine body, prostate, bladder, larynx, gallbladder, kidney, ureter of the kidney), by prefecture	54
95		Administrator	Health professionals	Number of cancer diagnoses compared to medical resources	Center, surgeon, specialist, medical specialist	12, 20, 42, 48
96		Administrator	Health professionals	Number of medical equipment (MRI, CT units, mammography, RT units, PET)	Per population (per 10,000, per 100,000), by region, by major, number of medical devices in laboratory	52
97		Administrator	Health professionals	Number of cancer patients receiving treatment/cancer surgery at regional general hospitals	Per 100,000 population	47, 48, 51, 54, 59, 62, 65
98		Administrator	Health professionals	Number of instruments for immunology and molecular analysis	Per million population	46, 47,
99		Administrator	Health professionals	Number of laboratories supporting screening, diagnosis and treatment needs	By region, hospitals and clinics	11, 13, 46, 49,
100		.Provider/Admin	Health professionals	Number of test centers per person	By region, hospitals and clinics	52
101		.Provider/Admin	Health professionals	The number and distribution of staff qualified to conduct screening services	By region, hospitals and clinics	52
102		Administrator	General health checkup	Health checkup rate	By region, hospitals and clinics	52

Sl. No	Classification	Participants	Subdomains	Indicators	Measurement	Reference
103	Secondary prevention	Administrator	Specific diagnosis & treatment	Scope of involvement in diagnosis (stage)	Conduction at diagnosis (local, small lymphatic resuscitation, adjacent organ infiltration, distant metastasis, unknown) distribution (%)	59
104		Administrator	Treatment /consultation/Interruption rate	Treatment rate (%) within the counting target area (diagnosis area)	By site (excluding or including intraepithelial cancer)	57
105		Provider	Patient-centered	Whether to request or test genetic counseling for high genetic risk factors for colorectal cancer	By age, sex, family history	48
106		Administrator	Patient centered	Number of cancer-related education and interventions in the population	Cancer type	47
107		Administrator	Patient-centered	Distribution of the proportion of the number of enrollments with notification of disease name at the start of initial treatment (20 years of age or older)	Facilities, prefectures (823 facilities) (by hospital type)	52
108	Treatment	Administrator	Prevalence	Primary cancer invasive ratio (extent of disease)	By cancer type, sex, and age	59
109		Provider	Health Care system	Time taken from cancer diagnosis test to start of treatment	Within 1 week, within 30-90 days, within 1 year, by cancer type	47
110		Provider	Health Care system	Average waiting time for female surgery	Medical records	52
111		Patient	Appropriate procedure and treatment	Response to Advanced Early-Stage Hodgkin's Lymphoma Treatment	Medical records evaluation (Stage based treatment)	49
112		Provider	Appropriate procedure and treatment	PET, CT or radionuclide bone scan within 59 days after diagnosis of breast cancer I/IIA/IIB	Evaluation of medical records	23, 48
113		Provider	Appropriate procedure and treatment	Whether or not stereotactic radiation therapy (SABR)	Lung cancer (for stage 1 patients who cannot operate surgery)	49
114		Provider	Appropriate procedure and treatment	Laboratory analysis rate of breast cancer immunohistochemistry	Estrogen receptor (ER), progesterone receptor (PR), epithelial growth factor receptor type 2 (IHC, FISH))	23, 48, 52
115		Provider	Appropriate procedure and treatment	Biopsy for diagnosis and treatment of prostate cancer	Male patients, Medical records and laboratory data analysis	49

Sl. No	Classification	Participants	Subdomains	Indicators	Measurement	Reference
116	Treatment	Provider	Appropriate procedure and treatment	Proportion of suitable surgery in early cervical cancer patients	Surgical records assessment [Total Abdominal Hysterectomy (TAH), Bilateral Salpingo Oophorectomy]	51, 16, 40
117		Provider	Appropriate procedure and treatment	Whether radioactive chemotherapy was performed for local advanced non-small cell lung cancer (stage 3)	Medical records of chemotherapy data analysis	31, 49
118		Provider	Appropriate procedure and treatment	Whether PET or PET-CT is prescribed within 12 months after treatment of patients with non-small cell cancer (stage 1, 2)	Medical records and laboratory data analysis	48
119		Provider	Appropriate procedure and treatment	Whether PET, CT, or radionuclide skeletal scans within 2 months after initial prostate cancer diagnosis with low risk of metastasis	Male patients, age, medical records and laboratory data analysis	31, 48
120		Provider	Appropriate procedure and treatment	Molecular testing of non-small cell stage IV patients with adenocarcinoma tissue	By cancer types, medical records and laboratory data analysis	48
121		Provider	Appropriate procedure and treatment	Molecular testing time required for non-small cell stage IV patients with adenocarcinoma tissue	By cancer types, medical records and laboratory data analysis	48
122		Provider	Appropriate procedure and treatment	BRCA1 and BRCA2 sequencing in epithelial ovarian cancer	By female population (within 1 month after birth)	24, 26, 51
123		Provider	Appropriate procedure and treatment	Whether a serotonin antagonist is prescribed or administered to chemotherapy patients at high risk/severe risk of vomiting	History of chemotherapy patients s data	48
124		Provider	Appropriate procedure and treatment	Prescribing or administering serotonin antagonists and corticosteroids to chemotherapy patients at high risk/severe risk of vomiting	By Cancer type , Chemotherapy patients' data	48
125		Provider	Appropriate procedure and treatment	Whether or not NK1 receptor antagonists and olanzapine are prescribed or administered to chemotherapy patients at high risk of vomiting.	By Cancer type , chemotherapy patients' data	48
126		Provider	Appropriate procedure and treatment	Whether NK1 receptor antagonists and olanzapine are prescribed or administered to first-line chemotherapy patients with low or moderate risk of vomiting	By Cancer type, Chemotherapy data	48
127		Provider	Appropriate procedure and treatment	Whether antiemetics are prescribed or administered to patients on chemotherapy with moderate risk of vomiting	Cancer type , Medication records	48
128		Provider	Appropriate procedure and treatment	Whether or not trastuzumab [herceptin] is recommended for patients with Her2/neu positive breast cancer stage 1 (T1c) to stage 3 (AJCC classification)	By Cancer type , Medication records	48
129		Provider	Appropriate procedure and treatment	Whether or not trastuzumab [herceptin] is administered to patients with Her2/neu positive breast cancer IA (T1c), IB to stage 3 (AJCC classification)	By Cancer type , Medication records	48
130		Provider	Appropriate procedure and treatment	Whether trastuzumab [herceptin] is administered to patients with Her2/neu-negative breast cancer or unrecorded	By Cancer type , Medication records	48

Sl. No	Classification	Participants	Subdomains	Indicators	Measurement	Reference
131		Provider	Appropriate procedure and treatment	Whether tamoxifen or an aromatase inhibitor was administered within 1 year after diagnosis to patients with ER or PR positive breast cancer IA (T1c), IB to stage 3 (AJCC classification)	By Cancer type , Medication records	48
132		Provider	Appropriate procedure and treatment	Whether or not CD antigen expression is negative or unrecorded patients are administered obinutuzumab, ofatumumab, or rituximab	By Cancer type , Medication records	48
133		Provider	Appropriate procedure and treatment	Platin or taxane within 42 days after cytoreduction to women with invasive stage 1 (grade3), ICIV ovarian, fallopian tube, or peritoneal cancer	By Cancer type , Medication records	48
134		Provider	Appropriate procedure and treatment	Whether to manage venous thromboembolism (VTE prophylaxis) within 24 hours after cell count reduction in women with invasive stage 1 (grade3), ICIV ovarian, fallopian tube, or peritoneal cancer	By Cancer type , Medication records	48
135		Provider	Appropriate procedure and treatment	Whether to perform early chest radiotherapy for patients diagnosed with limited stage small cell lung cancer (SCLC)	History of radiotherapy patients s data	31, 48
136		Provider	Appropriate procedure and treatment	Hepatitis B virus infection test (HBsAg) and hepatitis B core within 3 months of taking Obinutuzumab, ofatumumab, or rituximab in malignant non-Hodgkin's lymphoma (NHL) patients Anti-HBc test	Medication records	48
137		Provider	Appropriate procedure and treatment	Whether mammography and genetic tests are performed for breast cancer treatment	Medical records and special procedures data analysis	23, 28, 35, 39, 44, 48
138	Treatment	Provider	Appropriate procedure and treatment	Whether to perform imaging tests before colon cancer surgery (Imaging)	Medical records and special procedures data analysis	18, 27, 32, 51, 52, 59
139		Provider	Appropriate procedure and treatment	Cancer fetal antigen (CEA) levels within 4 months after colon cancer resection	Medical records and special procedures data analysis	48,
140		Provider	Appropriate procedure and treatment	Whether a genetic test for the management of patients with invasive colorectal cancer	Medical records and special procedures data analysis	27, 48
141		Provider	Appropriate procedure and treatment	BRAF status in patients with advanced melanoma (stage III, IV) incapable of surgical resection	Medical records and special procedures data analysis	47
142		Provider	Appropriate procedure and treatment	Presence or absence of chemotherapy for metastatic solid cancer patients with 3, 4, or undocumented systemic performance	Medical records and special procedures data analysis	49
143		Provider	Appropriate procedure and treatment	Content of resection (location and site)	By cancer type (breast cancer, prostate, stomach, colon, rectum, female breast, pancreas, cervix), by cancer stage	48
144		Provider	Appropriate procedure and treatment	Whether colonoscopy before or within 6 months after treatment	Medical records and special procedures data analysis	46-49,32-34
145		Provider	Appropriate procedure and treatment	RAS (KRAS and NRAS) testing for metastatic colorectal cancer patients who received anti-EGFR MoAb therapy	Medical records and special procedures data analysis	48

Sl. No	Classification	Participants	Subdomains	Indicators	Measurement	Reference
146		Provider	Appropriate procedure and treatment	Prophylactic Cranial Irradiation for Limited Stage SCLC	Medical records and special procedures data analysis	48
147		Provider	Appropriate procedure and treatment	Appropriate treatment for dyspnea	Medical records and special procedures data analysis	48
148		Provider	Appropriate procedure and treatment	Granulocyte colony-stimulating factor (GCSF) administration to patients receiving metastatic chemotherapy	By cancer type (breast cancer, colon cancer)	48
149	Treatment	Provider	Appropriate procedure and treatment	Whether or not chemotherapy has been completed	Medical records of chemotherapy data analysis	48
150		Provider	Appropriate procedure and treatment	Whether anti-EGFR MoAb therapy received in patients with KRAS and NRAS mutations	Medical records and special procedures data analysis	48
151		Provider	Appropriate procedure and treatment	Whether or not patients have undergone excessive treatment for chemotherapy using platinum	Medical records of chemotherapy data analysis	48
152		Provider	Appropriate procedure and treatment	Whether hormonal drugs are used	Medication records	48
153		Provider	Appropriate procedure and treatment	Whether Tamoxifen or Aromatase Inhibitor is recommended within 1 year after diagnosis to patients with ER or PR-positive breast cancer IA (T1c), IB to stage III (AJCC classification)	Medication records	48
154		Administrator	Appropriate procedure and treatment	Contents of ablation in the counted area (%)	By site (excluding or including intraepithelial cancer)	59
155		Administrator	Appropriate procedure and treatment	UICC TNM classification treatment method ratio by stage	By period (before or last), comprehensive treatment method (surgery only, endoscopy only, surgery + endoscopy, radiation only, drug treatment only, radiation + drugs, / endoscopy + radiation, surgery / endoscopy + drugs, surgery / endoscopy+ other, surgery/endoscope +radiation+ drugs, other combinations, no treatment), with or without treatment at other facilities	59
156		Administrator	Appropriate procedure and treatment	Initial treatment content rate (%)	By cancer type, excluding or including intraepithelial cancer, treatment (surgical, laparoscopic, endoscopic, radiation, chemotherapy, other treatments, no treatment)	59
157		Administrator	Appropriate procedure and treatment	Surgical, laparoscopic, and endoscopic treatment coverage (%)	By cancer type, treatment range (no residual tumor, with residual tumor, unknown), excluding or including intraepithelial cancer	59
158	Treatment	Administrator	Appropriate procedure and treatment	Maximum Surgical Resection (surgical resection- (≥90%* tumor))	By carcinoma (brain and central nervous system cancer, stomach cancer)	12, 13, 51,

Sl. No	Classification	Participants	Subdomains	Indicators	Measurement	Reference
159		Administrator	Appropriate procedure and treatment	Resection Margins Ratio	All cancer types, step by step	16, 17, 23, 28, 35
160		Administrator	Appropriate procedure and treatment	Percentage of patients who received PET or CT between 3 and 12 months after completing B-cell lymphoma treatment	Medical records and special procedures data analysis	48
161		Administrator	Appropriate procedure and treatment	Number of patients enrolled for radical radiation therapy	Cancer type	48
162		Administrator	Appropriate procedure and treatment	Number of patients receiving chemotherapy	By cancer type, by period	49
163		Administrator	Appropriate procedure and treatment	Number of patients hospitalized for cryotherapy after colposcopy	Cervical cancer, female, by region	47
164		Administrator	Appropriate procedure and treatment	Number of patients who received Neo-adjuvant Therapy before surgery	Rectal cancer	13, 34, 35, 51
165		Administrator	Appropriate procedure and treatment	The number of patients treated with anticancer chemotherapy and monoclonal antibody among follicular lymphoma patients	Lymphoma	47
166		Administrator	Appropriate procedure and treatment	Collecting data related to surgery-related activities	Surgical records assessment	52
167		Administrator	Appropriate procedure and treatment	Diagnosis rate of no metastatic rectal cancer patients who received pelvic MRI or ultrasound for staging prior to initial treatment or surgery	Medical records and special procedures data analysis	48
168		Administrator	Appropriate procedure and treatment	Proportion of patients who received male hormone blockade (ADT) for prostate cancer who received a bone density test to monitor bone loss within 1 year after starting ADT.	Male patients, age, duration of treatment, Medical records and special procedures data analysis	31, 48
169	Treatment	Patient/family/ Administrator	Appropriate procedure and treatment	Multidisciplinary treatment system rate	Regional based, hospitals	46
170		Provider/ Administrator	Appropriate procedure and treatment	Number of advanced cancer surgeries	Cancer type	13, 23, 24, 51
171		Patient	Treatment plan & Record	Whether there is a family history of cancer (direct family and siblings)	Diagnosis status, diagnosis age	48
172		Provider	Treatment plan & Record	Suitability for provision of professional training programs for inpatients	By cancer type, by treatment	46
173		Provider	Treatment plan & Record	Suitability of providing education for self-management and follow-up management for discharged patients	By cancer type, by treatment	46

Sl. No	Classification	Participants	Subdomains	Indicators	Measurement	Reference
174		Provider	Treatment plan & Record	Determination of cancer stage in patients with invasive breast cancer (cancer stage, HER2, ER/PR status)	Medical records and special procedures data analysis	46
175		Provider	Treatment plan & Record	Includes critical sessions in patient care planning (evaluation, goal, objectives, strategy, time planning, managers, resources, monitoring and evaluation)	Medical records assessment	48
176		Provider	Treatment plan & Record	Presence or absence of a documented plan for chemotherapy	Medical records of chemotheraphy data analysis	47
177		Provider	Treatment plan & Record	Patient Consent for Chemotherapy	Medical records of chemotheraphy data analysis	48
178		Provider	Treatment plan & Record	Protection of personal information and confidentiality related to cancer diagnosis, and whether consent for treatment is collected	By cancer type, by treatment procedure	48
179		Provider	Treatment plan & Record	Whether a summary of chemotherapy treatment is completed within 3 months of the end of chemotherapy treatment	Medical records of chemotheraphy data analysis	48
180		Provider	Treatment plan & Record	Pain treatment plan for cancer patients	Advanced, metastatic lung cancer, colon cancer, pancreatic cancer, prostate cancer, pain level (moderate, severe)	48
181		Provider	Treatment plan & Record	Presence of chemotherapy intention (treatment versus non-treatment) recorded before or within 2 weeks after administration	Medical records of chemotheraphy data analysis	48
182		Provider	Treatment plan & Record	Surgical records of residual disease within 48 hours of cytoreduction in patients with invasive ovarian cancer, fallopian tube or peritoneal cancer	Medical records and special procedures data analysis	48
183	Treatment	Provider	Treatment plan & Record	Whether advanced/metastatic lung cancer, pancreatic cancer, and colon cancer have been diagnosed within the first 3 visits, and whether to complete the Advance directive documentation	Medical records - by hospitals and cancer specialized center	48
184		Provider	Treatment plan & Record	Whether bone-modifying agents (IV bisphosphonates or denosumab) are administered to breast cancer bone metastasis	Medication records	48
185		Administrator	Treatment plan & Record	Integration of plans for chronic diseases and other related issues	By territory, by population group	46
186		Administrator	Treatment plan & Record	Treatment plan for chemotherapy according to management schedule	Dosing schedule (start date, treatment/rest period)	48
187		Patient	Treatment /consultation/Interruption rate	Systemic Therapy	By gender, year, cancer type, treatment	49
188		Administrator	Treatment /consultation/Interruption rate	Invasive (curative) treatment rate	By year (2013-2015), by cancer type (stomach cancer, colon liver, lung, female breast, cervix, prostate), by region	49

Sl. No	Classification	Participants	Subdomains	Indicators	Measurement	Reference
189		Patient/ provider /Admin	Treatment /consultation/Interruption rate	The number of registrations by the presence or absence of treatment at the own facility when initial treatment starts at the own facility.	By hospital type	55, 56
190		Provider	Patient-centered	Whether to discuss chemotherapy with the patient	Medical records of chemotheraphy	48
191		Provider	Patient-centered	Observation of constipation, fatigue, nausea, dyspnea, and vomiting in cancer patients	Metastatic lung cancer, pancreatic cancer, rectal cancer	52
192		Administrator	Patient-centered	Mental health and prevalence of psychosocial therapy	Medical records	48
193	Quality of life	Administrator	Survival/ Mortality	Disease-Free Survival Rate	Cancer care service census assessment (Survivor health data)	48
194		Administrator	Survival/ Mortality	Mortality rate	Sex, cancer type, age, year, region, age-adjusted mortality rate (per 100,000 population), cause of death (tuberculosis, cerebrovascular disease, malignant neoplasms, heart disease, senility, pneumonia),	47-48, 54-57, 60, 61
195		Administrator	Survival/ Mortality	Quality of care and survival training	By year, by cancer type, by region	46
196	Quality of life	Administrator	Survival/ Mortality	Estimate expected deaths among loss to follow-up	Mortality rate within 12 months after hospitalization as a cancer patient (cause of death-chronic heart failure)	46
197		Administrator	Survival/ Mortality	Death after surgery	Within 30 days, within 30-60 days, within 30-90 days, 1 year	49
198		Administrator	Survival/ Mortality	Number of deaths (absolute value)	By cancer type, by region	49, 56, 59
199		Administrator	Survival/ Mortality	Risk of death	By gender, age, and cancer type	55
200		Administrator	Survival/ Mortality	Standardized death ratio	By cancer type, by region	57
201		Administrator	Survival/ Mortality	Place of death of cancer patients	Medical facilities (internal/external)	52
202		Administrator	Survival/ Mortality	5-year relative survival rate	By gender, age, year, cancer type, survival year (1 year, 3 years, 5 years), by region, by clinical stage	52
203		Administrator	Survival/ Mortality	5-year observational survival rate (absolute survival rate)	By gender, age, year, cancer type, survival year (1 year, 3 years, 5 years), by region, by clinical stage	52

Sl. No	Classification	Participants	Subdomains	Indicators	Measurement	Reference
204		Administrator	Survival/ Mortality	Survivor's quality of life index	Surveys and interviews	49
205		Administrator	Survival/ Mortality	Potential Loss of Life Training (PYLL)	By cancer type, age, sex, family history	47, 48
206		Administrator	Prevalence	Cancer recurrence rate	By cancer type, by population group, by region	52
207		Administrator	Health care expenditure	Expenditure on medicines for cancer patients	Per patient, type of cancer	46
208		Administrator	Health care expenditure	Cancer treatment cost estimate	By region, by population group, by country	55
209		Administrator	Health care expenditure	Cancer treatment cost support priority/financial protection guaranteed	By region	52
210		Administrator	Health care expenditure	Medical cost high price index (compared to actual treatment cost high compared to the national average)	Cancer type	60
211		Administrator	Health care expenditure	Cancer-related rehabilitation costs	Cancer type	52
212	Quality of life	Administrator	Health Care system	Whether there is a support or regulatory policy for cancer patients	National wide and regional based	52
213		Administrator	Health Care system	Establishment and operation of an occupational cancer surveillance system (employment protection, insurance, and others)	National wide and regional based	47
214		Administrator	Health professionals	Completeness of cancer registration (process)	National wide and regional based	52
215		Administrator	Health professionals	Resources for radiation oncology (equipment per population, experts, etc.) (structure)	National wide and regional based	52
216		Administrator	Health professionals	Medical oncology resources (such as number of hospitals per day per population) (structure)	National wide and by region	52
217		Provider	Specific diagnosis & treatment	Compatibility of clinical guidelines and actual treatment	Hospitals and clinics (national cancer guidelines)	48
218		Provider	Specific diagnosis & treatment	Postoperative complications	By cancer type (stomach, colon, colon, rectum, prostate, bladder and renal ureter)	49

Sl. No	Classification	Participants	Subdomains	Indicators	Measurement	Reference
219		Provider	Specific diagnosis & treatment	Rate of additional gastrectomy after incomplete endoscopic resection	Medical records and special procedures data analysis	59
220		Provider	Specific diagnosis & treatment	ER/PR-negative breast cancer IA (T1c), IB to stage 3 (AJCC classification) For women under 70 years of age, whether or not combination chemotherapy is recommended within 4 months after diagnosis	Female population, age, medical history	48
221		Provider	Specific diagnosis & treatment	Among ER/PR-negative breast cancer IA(T1c), IB to stage 3 (AJCC classification), women under 70 years of age should receive combination chemotherapy within 4 months of diagnosis.	Female population, age, medical history, chemotherapy treatment history	48
222		Administrator	Treatment/ consultation/Interr uption rate	Follow-up dropout rate after diagnosis (Censored)	Total cancer, by cancer type (stomach, colon liver, lung, female breast, esophagus, pancreas, cervix, uterine body, prostate, bladder, larynx, gallbladder, kidney, renal ureter)	54
223		Patient	Patient-centered	Whether to consult a patient with invasive colorectal cancer or a genetic test result	Colon Cancer, Breast Cancer (7)	48
224		Administrator	Patient-centered	Percentage of cancer patients receiving social psychological treatment	by hospitals, region or public health centers	14, 50,
225		Administrator	Patient-centered	Waiting time more than 24 hours	Regional and hospitals	52
226	Survivor Man agement	Administrator	Health professionals	Whether human, financial and technical resources are appropriately allocated to support activities such as data collection and quality assurance of cancer registration.	Regional based census (general and cancer specialized hospital)	21, 35, 43-45, 49,
227		Administrator	Health Care system	Cancer patient medical expenses support	Medical records, national health insurance data, private insurance data	52
228		Administrator	Health Care system	Community-based cancer treatment service implementation	By age, childhood cancer, medical recipients	52
229		Provider	Specific diagnosis & treatment	Percentage of colorectal cancer patients who underwent PET or CT after completion of colorectal cancer treatment	Regional, Survivor-Centered Programs	48
230		Administrator	Specific diagnosis & treatment	Proportion of prostate cancer patients taking abiraterone receiving appropriate medications and monitoring	Medical records	31, 48
231		Administrator	Specific diagnosis & treatment	Proportion of patients with high/very high risk of recurrence prostate cancer who are prescribed adjuvant hormone therapy (GnRH antagonist) and receive external irradiation therapy.	Medical records	48
232		Provider	Treatment plan & Record	Documented daily performance records of patients with stage IV or distant metastatic non-small cell lung cancer (NSCLC)	Medical records	48

Sl. No	Classification	Participants	Subdomains	Indicators	Measurement	Reference
233		Patient	Patient-centered	Whether the patient's quality of life is improved before and after rehabilitation support	National cancer care survey (self-survey, interview) data	48
234		Patient	Patient-centered	The presence or absence of appropriately coped pain	Medical records	48
235		Provider	Patient-centered	Assessment and management of psychosocial pain	All, advanced or metastatic cancer (colorectal cancer, lung cancer, pancreatic cancer), by period (last 3 months)	14, 21, 32, 33, 36, 48
236		Provider	Patient-centered	Monitoring chemotherapy at the visit after the start of treatment	By cancer types, medical records	48 ,26, 38
237		Provider	Patient-centered	Discuss infertility risk with reproductive-age patients prior to chemotherapy	By cancer types, medical records	48
238		Administrator	Patient-centered	Percentage of patients with advanced cancer who assessed their quality of life each month	Medical records of regional and general hospitals	52
239		Administrator	Patient-centered	Social welfare services rate for survivors	By region per 1million population	52
240	End of life care	Administrator	Research &investment	Evidence-based research plans and priorities for palliative care	Number of articles published National and international level	24, 52
241	End of life c are	Administrator	Health professionals	Number of psychosocial therapists engaged in cancer management health care	By country, by region	46
242		Administrator	Prevalence	Whether to enroll in hospice before death	Death records (3 days before death, 7 days before, 2 months before death)	48
243		Administrator	Prevalence	Whether cancer patients have been registered for palliative care for the past 1 year	Yearly institution evaluation reports/data	52, 53
244		Administrator	Health Care system	Whether to conduct professional and formal evaluation of palliative care plans when treating cancer patients (for monitoring by decision makers (effectiveness, quality, and inclusiveness of palliative care))	Palliative center protocol assessment	49, 48, 52, 55, 56
245		Administrator	Health professionals	Whether or not to train palliative care provided by oncology specialist	Yearly. institution evaluation manpower and resource reports/data	46
246		Administrator	Health professionals	Number of palliative care professionals	Per organ, per cancer type, per patient	48
247		Administrator	Health professionals	The number of beds available at hospice palliative care institutions for terminally ill cancer patients	By palliative care centers	46

Sl. No	Classification	Participants	Subdomains	Indicators	Measurement	Reference
248		Administrator	Health professionals	Number of palliative care centers providing rehabilitation care services	Per regional center	46
249		Administrator	Health professionals	Whether to register for day care for terminally ill cancer patients	By hospital	46
250		Administrator	Health professionals	Resource existence/treatment availability of palliative care for children	By hospital	52
251		Administrator	Health professionals	The number of patients who received treatment in palliative care institutions	By hospital	47, 49, 52, 54
252		Provider	Specific diagnosis & treatment	Administration of chemotherapy for terminally ill patient at 2 weeks before death	Assessment of medical records	48
253		Patient	Patient centered	Institutional satisfaction survey for terminal cancer patients	Late evaluation, patient, family	14, 36, 46, 47
254		Provider	Patient centered	Pain evaluation system in terminal cancer patients	By cancer type, age, per 1000 people, by type of narcotic analgesics	46, 47, 53
